# The interactome of soluble amyloid‐β across the Alzheimer's disease continuum

**DOI:** 10.1002/alz70855_100163

**Published:** 2025-12-23

**Authors:** Dietmar Rudolf Thal, Xiaohang Li, Katerina Konstatoulea, Klara Gawor, Anne Wiedmer, Christine A.F. von Arnim, Rik Vandenberghe, Sebastien Carpentier, Markus Otto, Joost Schymkowitz, Frederic Rousseau, Jolien Schaeverbeke

**Affiliations:** ^1^ Laboratory of Neuropathology, KU Leuven, Leuven, Vlaams‐Brabant, Belgium; ^2^ CellCarta N.V., Wilrijk, Belgium; ^3^ Laboratory of Neuropathology, Leuven Brain Institute, KU Leuven, Leuven, Belgium; ^4^ Switch Laboratory, Leuven Brain Institute, KU Leuven, Leuven, Belgium; ^5^ UT Southwestern, Dallas, TX, USA; ^6^ University of Ulm, Ulm, Germany; ^7^ University Medical Center Göttingen, Göttingen, Germany; ^8^ University Hospitals Leuven, Leuven, Belgium; ^9^ Laboratory for Cognitive Neurology, KU Leuven, Leuven, Leuven, Belgium; ^10^ Laboratory of Tropical Crop Improvement, KU Leuven, Leuven, Belgium; ^11^ Department of Neurology, Ulm University Hospital, Ulm, Germany; ^12^ University Clinic and Polyclinic for Neurology, Martin‐Luther‐University Halle‐Wittenberg, Halle (Saale), Germany; ^13^ Laboratory for Cognitive Neurology, Leuven Brain Institute, KU Leuven, Leuven, Belgium

## Abstract

**Background:**

In Alzheimer's disease (AD), amyloid β (Aβ) aggregates, ranging from soluble Aβ oligomers to insoluble Aβ plaques. During Aβ aggregation, *in vitro* and *in silico* screening has shown that aggregation‐prone regions (APRs) appear as an optimal binding site for protein‐protein interactions. Interacting partners that define disease pathways provide potential insight into the cellular environment and protein interactions present during pathological Aβ aggregation. Using mass spectrometry, several proteins beyond Aβ have been discovered in AD pathogenesis. However, there is currently limited knowledge about proteins interacting with soluble Aβ in human brain tissue.

**Method:**

This study aimed to systematically assess which proteins interact with soluble Aβ in human cortex across the AD continuum (i.e. pathologically‐definite symptomatic AD, asymptomatic AD and non‐AD controls) through affinity purification‐mass spectrometry with a Aβ‐specific binding condition using two Aβ antibodies (4G8: Aβ_17‐24_ and 6E10: Aβ_1‐17_) and a non‐specific binding condition (magnetic beads‐bound proteins). For differential expression, Paired *T* tests were performed to detect proteins significantly more abundant in Aβ_17‐24_ and Aβ_1‐17_ binding conditions respectively, considered as the ‘’Aβ interactome’’. Co‐expression network analysis was performed to assess which proteins correlated with neuropathological staging. Proteins derived were then assessed for diagnostic group differences to detect early mediators of AD pathogenesis.

**Result:**

The combined Aβ_17‐24_ and Aβ_1‐17_ interactome consisted out of 129 proteins, including APOE and the glial fibrillary acidic protein and vimentin, which were significantly more abundant in Aβ‐IPs versus beads. Co‐expression analysis revealed a protein module which was significantly negatively correlated with Aβ phase severity. Diagnostic group comparison indicated that proteins involved in cytoskeletal modelling (septins), clathrin‐mediated endocytosis, chaperone function, and Rho GTPase activity were already reduced in the interactome of early stage AD cases. *In silico* and *in vitro* validation confirmed the existence of C‐terminal Aβ APR homology in 40% of the mass‐spectrometry derived Aβ interactome candidates (including PCSK1, HS105 and FN3K).

**Conclusion:**

Interactions of Aβ with proteins involved in septins, clathrin‐mediated endocytosis, and chaperone functions are diminished in AD, even in asymptomatic individuals. This observation suggests that the impaired physiological protein‐binding function of soluble Aβ might represent an early pathogenic event in the development of AD.

